# Supplementary material for: Comparative proteomics of Rhizopus delemar ATCC 20344 unravels the role of amino acid catabolism in fumarate accumulation
Source: PeerJ. 2017 Mar 30;5:e3133. doi: 10.7717/peerj.3133 (PMC5376114; doi:10.7717/peerj.3133)
Supplement: File S1 — Detailed description of RNA seq data processing and KEGG pathway mapping with all the tools and parameters used. [file peerj-05-3133-s001.docx]

## Data processing

In the following, we have provided one **example** command for every tool used for the RNA seq data processing, starting from the raw files deposited at the European Nucleotide Archive (http://www.ebi.ac.uk/ena/data/view/PRJEB14210).

1) Filtering ---------------------------------------------------------------------------------------------------

1.1) Getting rid of rRNA (.fastq and .log are added by default):

SortMeRNA v1.9

/home/programs/filtering/sortmerna-1.9-linux-64-bin/sortmerna -n 8 --db /home/programs/filtering/sortmerna-1.9-linux-64-bin/rRNA_databases/rfam-5.8s-database-id98.fasta /home/programs/filtering/sortmerna-1.9-linux-64-bin/rRNA_databases/rfam-5s-database-id98.fasta /home/programs/filtering/sortmerna-1.9-linux-64-bin/rRNA_databases/silva-arc-16s-database-id95.fasta /home/programs/filtering/sortmerna-1.9-linux-64-bin/rRNA_databases/silva-arc-23s-database-id98.fasta /home/programs/filtering/sortmerna-1.9-linux-64-bin/rRNA_databases/silva-bac-16s-database-id85.fasta /home/programs/filtering/sortmerna-1.9-linux-64-bin/rRNA_databases/silva-bac-23s-database-id98.fasta /home/programs/filtering/sortmerna-1.9-linux-64-bin/rRNA_databases/silva-euk-18s-database-id95.fasta /home/programs/filtering/sortmerna-1.9-linux-64-bin/rRNA_databases/silva-euk-28s-database-id98.fasta -a 12 --I Air24h3_AGTCAA_L008_R1_001_AC0RCJACXX.filt.fastq --accept ../01output_QC/Air24h3_AGTCAA_L008_R1_001_filt_rRNA --other ../01output_QC/Air24h3_AGTCAA_L008_R1_001_filt_mRNA --log ../01output_QC/Air24h3_AGTCAA_L008_R1_001_rRNAfiltering

1.2) Getting rid of adapters (note that adapter trimming was already done by illumina in-house pipeline, so this step is not necessarily needed):

cutadapt v1.2.1

/home/programs/filtering/cutadapt-1.2.1/bin/cutadapt $(</home/programs/filtering/cutadapt-1.2.1/truseq_-b.conf) ./01output_QC/Air24h3_AGTCAA_L008_R1_001_filt_mRNA.fastq > ./01output_QC/Air24h3_AGTCAA_L008_R1_001_filt_mRNA_cutadapt.fastq

1.3) General quality filtering:

PRINSEQ v0.20.2

perl /home/programs/filtering/prinseq-lite-0.20.2/prinseq-lite.pl -fastq ./01output_QC/Air24h3_AGTCAA_L008_R1_001_filt_mRNA_cutadapt.fastq -min_len 20 min_qual_mean 30 -trim_qual_left 30 -trim_qual_right 30 -ns_max_n 3 –noniupac

Output:

Air24h3_R1:

Input sequences: 19,629,663

Input bases: 991,157,607

Input mean length: 50.49

Good sequences: 19,579,237 (99.74%)

Good bases: 985,788,859

Good mean length: 50.35

Bad sequences: 50,426 (0.26%)

Bad bases: 778,979

Bad mean length: 15.45

Sequences filtered by specified parameters:

trim_qual_left: 8732

min_len: 39293

ns_max_n: 2401

Air24h3_R2:

Input sequences: 19,985,084

Input bases: 1,011,349,818

Input mean length: 50.61

Good sequences: 19,696,245 (98.55%)

Good bases: 987,058,290

Good mean length: 50.11

Bad sequences: 288,839 (1.45%)

Bad bases: 14,673,722

Bad mean length: 50.80

Sequences filtered by specified parameters:

trim_qual_left: 119126

min_len: 166921

ns_max_n: 2792

N24h3_R1:

Input sequences: 23,136,725

Input bases: 1,170,281,145

Input mean length: 50.58

Good sequences: 23,121,692 (99.94%)

Good bases: 1,165,137,130

Good mean length: 50.39

Bad sequences: 15,033 (0.06%)

Bad bases: 399,421

Bad mean length: 26.57

Sequences filtered by specified parameters:

trim_qual_left: 2153

min_len: 10018

ns_max_n: 2862

N24h3_R2:

Input sequences: 23,165,827

Input bases: 1,172,272,973

Input mean length: 50.60

Good sequences: 23,108,607 (99.75%)

Good bases: 1,161,659,627

Good mean length: 50.27

Bad sequences: 57,220 (0.25%)

Bad bases: 2,906,349

Bad mean length: 50.79

Sequences filtered by specified parameters:

trim_qual_left: 18677

min_len: 35319

ns_max_n: 3224

2) *De novo* assembly ---------------------------------------------------------------------------------------

2.1) Combining the reads:

cat ./01output_QC/Air24h3_AGTCAA_L008_R1_001_filt_mRNA_cutadapt_prinseq_good_D1q3.fastq ./01output_QC/Air24h3_AGTCAA_L008_R2_001_filt_mRNA_cutadapt_prinseq_good_V3Vm.fastq ./01output_QC/N24h3_AGTTCC_L008_R1_001_filt_mRNA_cutadapt_prinseq_good_2q0z.fastq ./01output_QC/N24h3_AGTTCC_L008_R2_001_filt_mRNA_cutadapt_prinseq_good_tw1w.fastq > ./01output_QC/Air24h3_N24h3_R1R2_QCcombinedReads.fastq

2.2) Assembly:

IDBA-UD assembler v1.1

idba_ud

/home/programs/assembly/idba-1.1.1/bin/idba_ud -r ./01output_QC/Air24h3_N24h3_R1R2_QCcombinedReads.fastq -o ./02output_QC_assembly/ num_threads 25

idba_tran

/home/programs/assembly/idba-1.1.1/bin/idba_tran -r ./01output_QC/Air24h3_N24h3_R1R2_QCcombinedReads.fastq -o ./02output_QC_assembly_idbaTran/ num_threads 25

Output idba-ud:

contigs: 13533 n50: 1419 max: 8567 mean: 1086 total length: 14703366 n80: 758

aligned 68268220 reads

Output idba-tran:

contigs: 17432 n50: 1584 max: 8567 mean: 1230 total length: 21450431 n80: 894

aligned 62802852

From here, we continued with the output from IDBA-UD only.

3) Read mapping --------------------------------------------------------------------------------------------

3.1) Own assembly:

Bowtie2 v 2.2.2

3.1.1) Indexing the reference file:

/home/programs/mapping/bowtie2-2.2.2/bowtie2-build -f ./02output_QC_assembly/contig.fa ./02output_QC_assembly/contig_bowtieBuild.fa

3.1.2) Mapping:

/home/programs/mapping/bowtie2-2.2.2/bowtie2 -x ./02output_QC_assembly/contig_bowtieBuild.fa -1 ./00raw_data/Air24h3_AGTCAA_L008_R1_001_AC0RCJACXX.filt.fastq -2 ./00raw_data/Air24h3_AGTCAA_L008_R2_001_AC0RCJACXX.filt.fastq -S ./03output_QC_mapping/Air24h3rawSequences_mappedAgainst_Air24h3N24h3QC_contig.sam -t -p 10

Output:

Aerobic:

20539199 reads; of these:

20539199 (100.00%) were paired; of these:

4991998 (24.30%) aligned concordantly 0 times

15285321 (74.42%) aligned concordantly exactly 1 time

261880 (1.28%) aligned concordantly >1 times

----

4991998 pairs aligned concordantly 0 times; of these:

95819 (1.92%) aligned discordantly 1 time

----

4896179 pairs aligned 0 times concordantly or discordantly; of these:

9792358 mates make up the pairs; of these:

8768966 (89.55%) aligned 0 times

961460 (9.82%) aligned exactly 1 time

61932 (0.63%) aligned >1 times

78.65% overall alignment rate

Anaerobic:

24519028 reads; of these:

24519028 (100.00%) were paired; of these:

6129005 (25.00%) aligned concordantly 0 times

18093953 (73.80%) aligned concordantly exactly 1 time

296070 (1.21%) aligned concordantly >1 times

----

6129005 pairs aligned concordantly 0 times; of these:

143702 (2.34%) aligned discordantly 1 time

----

5985303 pairs aligned 0 times concordantly or discordantly; of these:

11970606 mates make up the pairs; of these:

10708958 (89.46%) aligned 0 times

1171537 (9.79%) aligned exactly 1 time

90111 (0.75%) aligned >1 times

78.16% overall alignment rate

31.3) Converting the .sam file to a .bam file, then sorting and indexing the file:

samtools view -b -S ./03output_QC_mapping/Air24h3rawSequences_mappedAgainst_Air24h3N24h3QC_contig.sam -o ./03output_QC_mapping/Air24h3rawSequences_mappedAgainst_Air24h3N24h3QC_contig.bam

samtools sort ./03output_QC_mapping/Air24h3rawSequences_mappedAgainst_Air24h3N24h3QC_contig.bam ./03output_QC_mapping/Air24h3rawSequences_mappedAgainst_Air24h3N24h3QC_contig_sorted

samtools index ./03output_QC_mapping/Air24h3rawSequences_mappedAgainst_Air24h3N24h3QC_contig_sorted.bam

3.2) RA99-880 – output from the mapping:

Aerobic:

20539199 reads; of these:

20539199 (100.00%) were paired; of these:

6141069 (29.90%) aligned concordantly 0 times

11973016 (58.29%) aligned concordantly exactly 1 time

2425114 (11.81%) aligned concordantly >1 times

----

6141069 pairs aligned concordantly 0 times; of these:

21917 (0.36%) aligned discordantly 1 time

----

6119152 pairs aligned 0 times concordantly or discordantly; of these:

12238304 mates make up the pairs; of these:

10352813 (84.59%) aligned 0 times

1629658 (13.32%) aligned exactly 1 time

255833 (2.09%) aligned >1 times

74.80% overall alignment rate

Anaerobic:

24519028 reads; of these:

24519028 (100.00%) were paired; of these:

6642150 (27.09%) aligned concordantly 0 times

14799934 (60.36%) aligned concordantly exactly 1 time

3076944 (12.55%) aligned concordantly >1 times

----

6642150 pairs aligned concordantly 0 times; of these:

28792 (0.43%) aligned discordantly 1 time

----

6613358 pairs aligned 0 times concordantly or discordantly; of these:

13226716 mates make up the pairs; of these:

10908367 (82.47%) aligned 0 times

2006935 (15.17%) aligned exactly 1 time

311414 (2.35%) aligned >1 times

77.76% overall alignment rate

4) Coverage calculation ------------------------------------------------------------------------------------

BEDtools (note the genome file required is tab delimited and structured as follows: <chromName><TAB><chromSize>. This file was created using an own Python script, using the contig.fa output files obtained from the assembly step above as input)

/home/programs/genomics-assembly-misc/bedtools-2.17.0/bin/genomeCoverageBed -d -ibam ./03output_QC_mapping/Air24h3rawSequences_mappedAgainst_Air24h3N24h3QC_contig_sorted.bam -g ./04output_QC_coverage/AirN_QCcontig_genome.txt > ./04output_QC_coverage/Air_QCcontig_bedCoverage.txt

The average nucleotide coverage per contig was calculated from the output files generated above with another Python script.

Once EC numbers are assigned to the predicted proteins using PRIAM (see reference in main manuscript), they can be used to group into KEGG (see reference in main manuscript) defined pathways. The corresponding EC numbers for any given KEGG pathway can be found under: http://rest.kegg.jp/get/<ecpathwaymap>

**Example: Arginine biosynthesis (map:00220)**

http://rest.kegg.jp/get/ec00220

File from KEGG:

ENTRY ec00220 Pathway

NAME Arginine biosynthesis

CLASS Metabolism; Amino acid metabolism

PATHWAY_MAP ec00220 Arginine biosynthesis

MODULE M00028 Ornithine biosynthesis, glutamate => ornithine [PATH:ec00220]

M00029 Urea cycle [PATH:ec00220]

M00763 Ornithine biosynthesis, mediated by LysW, glutamate => ornithine [PATH:ec00220]

DBLINKS GO: 0006525

ENZYME 1.14.13.165

1.14.13.39

1.2.1.-

1.2.1.38

1.4.1.2

1.4.1.3

1.4.1.4

2.1.3.3

2.1.3.9

2.3.1.1

2.3.1.35

2.6.1.-

2.6.1.1

2.6.1.11

2.6.1.2

2.7.2.-

2.7.2.2

2.7.2.8

3.5.1.14

3.5.1.16

3.5.1.2

3.5.1.38

3.5.1.5

3.5.1.54

3.5.3.1

3.5.3.6

4.3.2.1

6.3.1.2

6.3.2.-

6.3.4.16

6.3.4.5

6.3.4.6

COMPOUND C00011 CO2

C00014 Ammonia

C00025 L-Glutamate

C00026 2-Oxoglutarate

C00049 L-Aspartate

C00062 L-Arginine

C00064 L-Glutamine

C00077 L-Ornithine

C00086 Urea

C00122 Fumarate

C00169 Carbamoyl phosphate

C00327 L-Citrulline

C00437 N-Acetylornithine

C00624 N-Acetyl-L-glutamate

C01010 Urea-1-carboxylate

C01250 N-Acetyl-L-glutamate 5-semialdehyde

C03406 N-(L-Arginino)succinate

C04133 N-Acetyl-L-glutamate 5-phosphate

C15532 N-Acetyl-L-citrulline

C20948 LysW-L-glutamate

C20949 LysW-L-glutamyl 5-phosphate

C20950 LysW-L-glutamate 5-semialdehyde

C20951 LysW-L-ornithine

KO_PATHWAY ko00220

///
